# Leukemia Inhibitory Factor: Roles in Embryo Implantation and in Nonhormonal Contraception

**DOI:** 10.1155/2014/201514

**Published:** 2014-07-24

**Authors:** Naguib Salleh, Nelli Giribabu

**Affiliations:** Department of Physiology, Faculty of Medicine, University of Malaya, 50603 Kuala Lumpur, Malaysia

## Abstract

Leukaemia inhibitory factor (LIF) plays an indispensible role in embryo implantation. Aberrant LIF production is linked to implantation failure. LIF regulates multiple processes prior to and during implantation such as uterine transformation into a receptive state, decidualization, blastocyst growth and development, embryo-endometrial interaction, trophoblast invasion, and immune modulation. Due to its critical role, LIF has been a target for a nonhormonal contraception. In this review, we summarize up-to-date information on the role of LIF in implantation and its role in contraception.

## 1. Introduction

Leukemia inhibitory factor (LIF), a pleiotropic cytokine from interleukin- (IL-) 6 family, regulates various cellular functions via binding to membrane-bound LIF receptor (LIFR) and gp130 [1]. Currently, three spliced variants of LIF have been identified which include membrane-associated, diffusible, and truncated forms acting as paracrine factors in embryo implantation [2]. Binding of LIF to LIFR recruits gp130 to form high affinity functional receptor complex leading to activation of downstream signal transduction pathway such as signal transducer and activator of transcription (STAT) [3]. In addition to the membrane-bound receptor, a number of soluble forms of LIF receptor have been identified which are involved in either potentiating or dampening LIF activities. The soluble forms of LIFR and gp130 can function as antagonists that compete with membrane-bound receptor for the binding to LIF [4]. Meanwhile, suppressor of cytokine signaling 3 (SOCS3) can also inhibit LIF signaling and can act as a negative regulator for LIF action [5]. Following binding of LIF to LIFR, SOCS3 inhibits LIF action via JAK1-STAT3 signaling pathway [6]. SOCS3 can also attenuate other signaling cascades which are induced upon LIF binding to LIFR and gp130 such as ERK-MAPK signaling pathway [7]. Few studies have demonstrated that LIF, gp130, and STAT are crucial for embryo implantation. Failure of blastocyst to implant has been reported in LIF gene knockout mice [8]. Meanwhile, mice with gp130 mutation and STAT-binding site deletion are also infertile indicating that gp130 and STAT are essential in regulating LIF action [3]. In species such as mice, uterine LIF displays biphasic expression pattern with the first peak appearing in the glands in preparation for uterine receptivity while the second peak appears in the stroma surrounding the implanting blastocyst at the time of attachment reaction [9]. In parallel, LIFR and gp130 are expressed in the luminal epithelia and stroma throughout the peri-implantation period [9] which further reinforce the critical role of LIF in embryo implantation.

Ovarian steroids are reported to play important role in regulating LIF, LIFR, and gp130 expressions in the uterus throughout the implantation window period. In mice, endometrial LIF secretion can be induced by nidatory estrogen at day 4 of pregnancy [10] while exogenous estrogen and progesterone administration to ovariectomised mice were able to increase gp130 expression in the uterine glands [11]. However, in humans, a report has indicated that luteal estrogen was not required to initiate the implantation process [12]. In hamsters, LIF secretion was induced by estrogen while the expression of LIFR and gp130 was induced by progesterone [12]. Currently, there is limited information with regard to regulations of LIF, LIFR, and gp130 expression in humans. An* in vitro* study using human endometrial stromal cell line indicated that concomitant administration of estrogen and progesterone was able to upregulate LIF receptor mRNA expression [13]. In humans, chorionic gonadotrophins (hCG) was also reported to upregulate LIF expression [14]. hCG and transforming growth factor- (TGF-) *β* increase LIF secretion by the cultured endometrial epithelial cells derived from follicular and secretory phases of the menstrual cycle [15]. Meanwhile, male seminal fluid was also found to stimulate LIF secretion by human endometrial epithelial cells* in vitro* [16].

Several strands of clinical evidences indicated important role of LIF during human embryo implantation. A moderate to high LIF expression was detected during the proliferative and secretory phases of the menstrual cycle in normal fertile women with low expression observed in infertile women with implantation failure. However, no differences in endometrial expression of gp130 were noted between fertile and infertile women [17]. Further assessment of uterine luminal fluid indicated that endometrium of infertile women secretes significantly lesser amount of LIF and gp130 than normal fertile women [18] between luteal days (LH) 6 to 13 which coincides with implantation window period [19].

**Figure 1:**
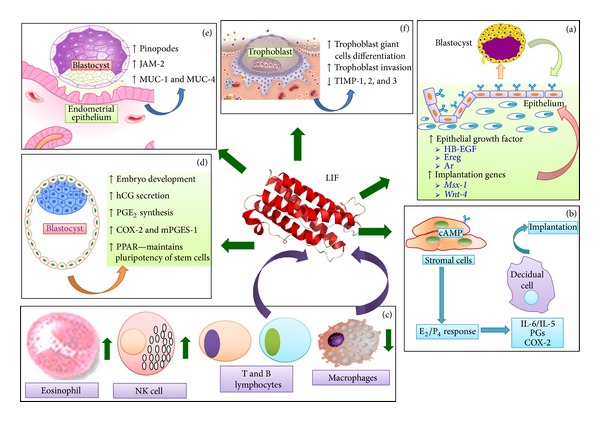
Summary of the known roles of LIF in embryo implantation. LIF increases the expression of EGF and implantation genes in receptive endometrium. LIF produced by endometrium and blastocyst regulates growth and development of the embryo. Meanwhile, LIF stimulates stromal decidualization by increasing the production of cytokines and prostaglandins. LIF is also involved in enhancing embryo-endometrial interaction through pinopodes and adhesion molecules. LIF stimulates trophoblast cells differentiation and increase trophoblast capability to invade the uterine stroma. Finally, LIF is involved in recruitment of specific cohort of leucocytes which participates in inflammatory response during implantation. LIF: leukemia inhibitory factor, HB-EGF: heparin binding-epidermal growth factor, Ereg: epiregulin, Ar: amphiregulin, E2: estrogen, P4: progesterone, IL: interleukins, PGs: prostaglandins, COX: cyclo-oxygenase, NK: natural killer, PPAR: peroxisome proliferator-activated receptor, PGE2: prostaglandins E2, hCG: human chorionic gonadotrophin, MUC: mucin, JAM: junctional adhesion molecules.

Evidences have shown that LIF is involved in the following events during implantation which include (i) endometrial transformation into a receptive state [2], (ii) embryo-endometrial interaction [20], (iii) stromal decidualization [21], (iv) trophoblast invasion [22], (v) blastocyst growth and development [8], and (vi) uterine leukocyte infiltration [13]. LIF has also been found to play an important role in regulating synthesis of prostaglandins (PGs), an important mediator of implantation and decidualization [23]. This review summarizes the current knowledge on the role of LIF in embryo implantation which could be used to guide further research in this field. Additionally, potential application of LIF as a target for nonhormonal contraception was also discussed.  Figure 1 summarizes the role of LIF in multiple steps during embryo implantation and placentation.

## 2. LIF Role in Uterine Transformation into a Receptive State

At the beginning of implantation window period in human, the expression of chicken ovalbumin upstream promoter transcription factor (COUP-TF) II, which is encoded by* NR2F2* gene [24] was increased in uterine stroma under the influence of progesterone [25]. This increase will result in suppression of uterine luminal epithelial cell proliferation via inhibition on estrogen receptor- (ER-) *α* activity [26]. Meanwhile, another endometrial transcription factor, Hand2, which was upregulated by progesterone also inhibits fibroblast growth factor- (FGF-) induced epithelial cell proliferation via downregulating ER-*α* expression and ERK1/2 signaling pathway in uterine luminal epithelia [27]. The role of LIF in the inhibition of epithelial proliferation at the onset of uterine receptivity period remains elusive. During uterine, receptivity, several changes in protein expression have been reported to occur in the uterine luminal epithelia which include increased synthesis of epithelial growth factor (EGF), for example, heparin-binding epidermal growth factor (HB-EGF) and its receptors, ErbB1 and ErbB2 [28]. In addition, increased expression of cytokines [18, 29] and intercellular adhesion molecules such as ICAM and fibrinogen-*γ* (FGG) has also been documented during this period [30].

LIF prepares the endometrium for embryo implantation. Several reports have indicated that in mice, peak expression of LIF occurs in the glands at the time of ovulation and prior to the onset of implantation [31, 32]. Epithelial-derived LIF was reported to act as autocrine regulator in the preparation of endometrium for implantation [8]. Female mice lacking of LIF gene suffered from implantation failure [32]. Meanwhile in humans, LIF expression in the endometrium was restricted to the glands, which was the highest during midluteal phase of the cycle [33]. In fertile women, LIF was also detected in uterine luminal fluid during the luteal phase of the menstrual cycle [14] and at the expected time of implantation [34]. In parallel, expression of LIFR-*β* was reported to be the highest in the luminal epithelia during secretory phases of the menstrual cycle while expression of gpl30 was found both in the luminal and glandular epithelia throughout menstrual cycle phases [20].

During receptivity period, LIF either binds directly to LIFR which is expressed on the blastocyst [35] or endometrial surfaces, in which the latter participates in autoregulation of LIF secretion [4]. LIF affects synthesis of growth factors in the endometrial epithelia. In LIF-deficient female mice, EGF-like growth factors such as amphiregulin (Ar), heparin binding epidermal growth factor (HB-EGF), and epiregulin (Ereg) were not expressed at the site of blastocyst apposition [36], although expressions of EGF receptors were not affected [36]. The dependency of Ar on LIF was evident from lack of expression of this growth factor in uterine luminal epithelia following administration of inhibitor to LIF (hLIF-05) [37]. LIF was also required to induce expression of implantation genes including Msx-1 and Wnt-4 [38]. Despite of these effects, direct role of LIF in regulating expression of adhesion molecules such as L-selectins, E-cadherins [39] and tight junction proteins, for example, claudin and occludin [40] which are expressed in the receptive endometrium remains elusive.

## 3. LIF Role in Decidualization

During the luteal phase of menstrual cycle and diestrus stage of oestrous cycle, stromal cells proliferate and differentiate into decidual cells which then produced various factors that help to prepare endometrium for blastocyst adhesion and subsequently trophoblast invasion. CCAAT/enhancer-binding protein *β* (C/EBP*β*) is a transcription factor that has been identified as a regulator of uterine stromal cell proliferation and differentiation in mice [41] and humans [21]. C/EBP*β* controls proliferation of primary human endometrial stromal cells (HESCs)* in vitro* by regulating expression of several key cell cycle-regulatory factors [42]. C/EBP*β* also increases the response of HESCs to estrogen, progesterone, and cyclic AMP (cAMP) and regulates interleukin- (IL-) 11 receptor and its downstream STAT3 transcription factor expression [21]. Female mice lacking C/EBP*β* gene are infertile with their uteri irresponsive towards deciduogenic stimuli while proliferation and differentiation of stromal cells were also impaired [43].

LIF plays important role in decidualization. Failure of stromal cells to differentiate into primary decidual cells has been reported in LIF-deficient mice [44]. LIF also enhances estrogen and progesterone-induced decidualization in HESCs via STAT3 phosphorylation [13]. Meanwhile, LIF was also found to upregulate the secretion of IL-6 and IL-15 from decidualized HESCs* in vitro* [13]. During decidualization, SOCS3 protein is stimulated in response to cytokine-induced STAT3 phosphorylation which acts as a negative-feedback inhibitor to hinder LIFR activity [45]. LIF was reported to indirectly stimulate the synthesis of PGs which is an important mediator of decidualization via IL-1 [46] and is required for cyclooxygenase-2 (COX-2) expression, in which the latter is a rate-limiting enzyme in the PGs synthesis [36]. Female mice lacking LIF gene suffered from implantation failure due to impaired PGs synthesis [36].

## 4. LIF Role in Leukocyte Recruitment during Implantation

In early pregnancy, infiltration of immune cells such as dendritic cells (DC), macrophages, T and B lymphocytes, natural killer (NK) cells [47], and neutrophils and eosinophils [48] into the endometrium was initially stimulated by factors in the seminal fluid [49] and later by the implanting blastocyst [50]. DCs are involved in immune tolerance, tissue remodeling, angiogenesis, and development of T regulatory (Treg) cells [51]. In humans, primary unexplained infertility was found to be associated with reduced expression of Treg in the endometrial tissue [52]. Macrophages participate in the progression of inflammation, counteract nitric oxide synthesis, tissue remodeling, angiogenesis, and immune tolerance towards the implanting blastocyst [53]. Meanwhile, T cells produced type-1 and type-2 cytokines which are involved in proinflammatory and anti-inflammatory responses in which changes in their ratio would determine the success of implantation [54]. In the late secretory phase and in early pregnancy, percentage of endometrium/decidual NK cells increases rapidly reaching up to 70% of the total uterine leukocyte population [55]. However, following implantation, endometrial NK cells differentiate into decidual NK cells, which begin to secrete cytokines (TNF-*α*, IL-10, GM-CSF, IL-1*β*, TGF-*β*1, CSF-1, LIF, and IFN-*γ*), growth factors, angiogenic factors as well as being involved in tissue remodelling, trophoblast migration, and decidualization [56].

LIF was reported to play important role in the regulation of immune response in the uterus in early pregnancy. LIF affects uterine leukocyte subpopulation and recruits specific cohort of leucocytes to the site of implantation [57]. LIF mRNA is expressed in decidual leucocytes itself [58]. LIF-deficient mice were found to have increased number of uterine macrophages although the number of NK cells and eosinophils [48] was reduced. Macrophage-derived LIF facilitates development of implantation-receptive endometrium by modulating the surface glycan structure of epithelial cells [59] as well as regulating the expression of fucosyltransferase enzyme in the uterine epithelia which is involved in the synthesis of embryo adhesive fucosylated glycoconjugates during the period of inflammatory response towards insemination [60]. A recent study reported that intrauterine administration of peripheral blood mononuclear cells (PBMCs) in mice helped to improve endometrial receptivity as evidence from a high pregnancy rate associated with increased endometrial LIF and vascular endothelial growth factor (VEGF) expressions [61]. PBMCs were found to produce cytokines and angiogenic factors necessary for implantation [56]. A study in humans has indicated that intrauterine infusion of PBMCs could help to improve the clinical pregnancy rate in patients with repeated implantation failure during* in vitro* fertilization-embryo transfer (IVF-ET) procedure [62], indicating that leucocytes play important role in ensuring the success of human embryo implantation.

## 5. LIF Role in Blastocyst Growth and Development

Following fertilization, embryo divides from 2- to 4-cell stages and subsequently 8-cell stage, in which the latter formed a morula which then develops into blastocyst that hatches upon entering the uterine cavity [63]. Blastocyst is then brought closer to the uterine wall by a force generated from fluid reabsorption in the uterine glands [64]. During blastocyst development, pluripotent inner cells are prepared for specific differentiation while outer trophectoderm cells interact with uterine epithelium in preparation for trophoblast invasion [65]. Mouse blastocysts were reported to express LIF mRNA transcript [66] which helps to increase rate of preimplantation embryo development [67, 68]. Meanwhile, mouse [69], rabbit [70] and human [71] blastocysts expressed LIFR and gp130 where the latter promotes preimplantation human embryo development in culture [72]. A combined administration of insulin-like growth factor- (IGF-) I, *β*-fibroblast growth factor (FGF), transforming growth factor- (TGF-) *β*1, granulocyte-monocyte colony stimulating factor (GM-CSF), and LIF have been reported to accelerate blastocyst development* in vitro*, especially changes from expanded to hatched blastocyst stages [73].

Leptin, a hormone linked to fertility acted via LIF to cause increased proportion of hatched blastocysts while causing decreased rate of embryo cell apoptosis* in vitro* via STAT3 signaling pathway [74]. LIF was found to affect hCG secretion by the trophoblast cells* in vitro* [75]. LIF was also reported to induce prostaglandin E (PGE_2_) production by human trophoblast cell line via stimulating COX-2 and microsomal PGE synthase-1 (mPGES-1) enzymes expression that are involved in PGE synthesis [23]. Meanwhile, LIF maintains pluripotency of mouse embryonic stem cells in culture via stimulating peroxisome proliferator-activated receptors (PPARs), a nuclear receptor transcription factors that regulates LIF-induced growth and self-renewal via tyrosine kinase 2-STAT3 signaling pathway [76].

## 6. Role of LIF in Embryo-Endometrial Interaction

During apposition phase of implantation, blastocyst initiates loose physical contact with the receptive endometrium which occurs prior to firm adhesion onto the endometrial surface. Mucin-1 (MUC-1), a glycocalyx which is expressed at the apical membrane of luminal epithelia, prevents firm blastocyst attachment [39]. At the site of trophoblast invasion, MUC-1 expression was markedly reduced [77]. Lack or aberrant MUC-1 production was reported to be one of the reasons of ectopic pregnancy [78]. MUC-1 provides scaffold for L-selectin ligand, which binds L-selectin on the blastocyst surface, allowing loose physical contact between blastocyst and endometrium as well as facilitating blastocyst rolling over the endometrial surface [79]. Meanwhile, L-selectin ligand, which is expressed on the pinopode (uterodomes) surface [80], also helps in blastocyst rolling. Finally, increased expression of other adhesion molecules such as *α*v*β*3 integrin [81], trophinins [82], junctional adhesion molecule (JAM) [83], and HB-EGF/errB4 complex [84] resulted in the blastocyst movement to come to a standstill which allows the blastocysts to firmly attach onto the endometrial surface prior to uterine invasion.

LIF plays indispensable role in initiating embryo-endometrial interaction. In mice lacking of LIF, absence of pinopodes was observed [44]. Meanwhile, human endometrium with uterodomes at different stages of development had both luminal and glandular epithelia expressing high levels of LIF and LIFR in the luteal days 6 through to 9 [85]. A study has shown that expression of LIFR and gp130 in the endometrium of fertile women positively correlated with pinopode formation, while the opposite was observed in women with unexplained infertility. A reduced level of LIF has been reported in hydrosalpinx [86] but not in recurrent pregnancy loss [87]. LIF, involving STAT3 phosphorylation, was reported to induce the expression of JAM-2 adhesion molecule which mediates the interaction between hatched blastocyst and receptive endometrium between days 3 and 4 of pregnancy in mice [83]. Although LIF effect on the expression of other adhesion molecules remains elusive, synchronous reduction in the levels of LIF, integrin *β*3, and MUC-1 was observed in the uterus of patients with hydrosalpinx [86], suggesting that the expression of these molecules was interdependent. Meanwhile, limited observation indicated that LIF affects expression of genes that encode antiadhesive mucins, MUC-1 and MUC-4 [60]. Other than these findings, LIF involvement in facilitating embryo-endometrial interactions remains to be fully elucidated.

## 7. Role of LIF in Trophoblast Invasion

Trophoblast giant cells, the first cell lineage derived from trophoblast stem cells [88], have the ability to invade into the decidua to initiate the implantation reaction [89]. As it moves towards the uterine compartment, trophoblast cells are confronted by various extracellular matrix (ECM) proteins and basement membranes such as collagen, fibronectin, laminin, vitronectin, trophin, and tastin which are able to bind to integrins on the trophoblast surface. These molecules help in controlling adhesion, migration, differentiation, and spreading of the trophoblast cells [90]. Invasion involves degradation of extracellular matrix (ECM) elements in the direction of migration which requires involvement of protease enzymes, such as matrix metalloproteinases (MMPs) 2, 9, and 14 [22] and is controlled by tissue inhibitor of metalloproteinases (TIMPs), for example, TIMPs 1, 2, and 3 [91].

LIF plays an important role in trophoblast invasion. LIF stimulates differentiation of trophoblast giant cells via JAK1-STAT3 pathway [6]. Meanwhile, soluble LIF provides extracellular signal that stimulates trophoblast invasion via STAT3 activation [92, 93]. LIF induces trophoblast cell proliferation via stimulating cell transition into G(2)/M phase of the cell cycle and activates both STAT3 and ERK1/2 signaling cascades [94]. Recently, LIF has been shown to increase invasiveness of human trophoblast cell line (HTR-8/SVneo cells)* in vitro* via STAT1 and STAT3 activation [95] as well as increase invasiveness of extravillous trophoblast cells via stimulating adhesion to the extracellular matrix elements including fibronectin, vitronectin, and laminin [96]. On the other hand, LIF was also reported to downregulate the expression of genes that encode TIMP1, TIMP2, and TIMP3 [95], therefore helping to reduce the expression of enzymes that are involved in potentiating trophoblast invasion. LIF was also reported to decrease the expression of integrin *β*_4_ mRNA in the trophoblast cells which promotes trophoblast invasion [96].

## 8. LIF as a Target for Nonhormonal Contraception

A study in humans indicated that low concentration of LIF in the maternal plasma was associated with increased risks of early pregnancy loss during embryo transfer [97], which points towards critical role of LIF in ensuring the success of embryo implantation. However, despite of this report, administration of recombinant LIF during assisted reproductive techniques (ART) has revealed no improvement in implantation rates in women with recurrent unexplained implantation failure [98]. Meanwhile, LIF has been identified as a potential target in the development of nonhormonal birth control vaccine. A study in mice indicated that intraperitoneal injection of anti-LIF antibody inhibited embryo implantation [99] while immunization of female mice with LIF or LIFR peptide vaccines induced long-lasting antibody development which could block fertility [100]. A preliminary study in rhesus monkey indicated that administration of monoclonal anti-LIF antibody could prevent embryo implantation [101].

In mice, intraperitoneal administration of LIF antagonist (LA) alone or conjugated to polyethylene glycol (PEGLA) between days 2.5 and 3.5 of pregnancy resulted in implantation failure which demonstrates that this compound could effectively be used as a nonhormonal contraceptive agent that targets LIF signaling in the endometrium [102]. Recently, vaginal administration of PEGLA in mice has been proven to be effective in inhibiting embryo implantation [103] while systemic administration of this compound to cynomolgus monkey reduced endometrial STAT3 phosphorylation, inhibited LIF-induced expression of cochlin, insulin-like growth factor-binding protein- (IGF-BP-) 3, vascular endothelial growth factor- (VEGF-) A, and COX-2 enzyme which are essential for embryo implantation [104]. While most works related to the use of LIF antagonist and PEGLA as nonhormonal contraceptive agents were preliminary and were limited to animal studies, a study using human tissue has been recently performed* in vitro* by Lalitkumar et al. [105]. In this study, the effect on human embryo attachment rate, embryo quality, and blastocyst expression of cell survival factor (Akt) and caspase-3 following exposure to endometrial tissue collected at luteal day 4 (LH+4) treated with PEGLA were determined. The findings indicated that in tissues treated with PEGLA, embryo attachment rate was reduced with embryonic LIF triggered apoptosis being inhibited. Meanwhile, endometrial LIF expression was also downregulated which was associated with the reduction in blastocyst survival rate and the increase in caspase-3 expression in the blastocyst. Currently, no clinical trials have been conducted in humans to assess effectiveness of LA or PEGLA as nonhormonal contraceptive agents.  Table 1 summarizes the studies performed using various models to investigate the effectiveness of LA or PEGLA as potential nonhormonal contraception agents.

## 9. Perspective

LIF is undoubtedly important in embryo implantation in rodents, primates, and humans. LIF has been shown to mediate multiple processes of embryo implantation ranging from blastocyst growth and development, uterine preparation for implantation, decidualization, uterine inflammatory responses towards the implanting embryos, embryo-endometrial interaction, and trophoblast invasion. In view of these documented roles, LIF has been proposed as a potential target for nonhormonal contraception. While most information with regard to the mechanisms underlying LIF actions in uterus during implantation period was obtained mostly from studies involving rodents and endometrial cell lines, more works are needed in humans to elicit its role in blastocyst implantation.

## Figures and Tables

**Table 1:** Summary of the literatures that reported the use of LIF antagonist in preventing implantation in different models. So far, only one study has been performed on human uterine tissues *in*-*vitro* which investigated this effect.

Authors	Antagonist	Route of administration	Detectable in uterine tissue	Model	Effects
Aschenbach et al. (2013) [104]	PEGLA	Intramuscular and subcutaneous	Yes luminal and glandular epithelia; endometrial lysates (intramuscular administration)	Cynomolgus monkeys	Reduced endometrial STAT3 protein phosphorylation *in vivo* and *in vitro* Inhibited LIF induced expression of cochlin, IGF-BP 3, VEGF A, and COX-2 in endometrial explants *in vitro *
		Vaginal	No		

Menkhorst et al. (2011) [103]	PEGLA	Vaginal	Yes (no systemic side effects)	Mice	Blocked implantation

White et al. (2007) [102]	LIF antagonist (LA)	Intraperitoneal plus continuous administration via miniosmotic pump		Mice	Blocked implantation Reduced STAT3 phosphorylation in luminal epithelial cells
	PEGLA	Intraperitoneal	Yes; uterine luminal epithelium (no systemic side effects)	Mice	Inhibited implantation Reduced STAT3 phosphorylation in luminal epithelial cells

Lalitkumar et al. (2013) [105]	PEGLA	*In*-*vitro* study on timed human endometrial biopsy tissue	Yes	Human	Reduced embryo attachment rate to endometrium Decreased LIF mRNA and protein in endometrium Inhibition of embryonic LIF triggered endometrial cell apoptosis Downregulation in AKT activation and increase of caspase-3 activation in blastocysts

Sengupta et al. (2006) [101]	Anti-LIF monoclonal Ab	Intrauterine	Yes	Rhesus Monkey	Significant decline in pregnancy outcome
